# Acute Renal Failure in a Patient With Severe Acute Respiratory Syndrome Coronavirus 2

**DOI:** 10.7759/cureus.13406

**Published:** 2021-02-17

**Authors:** Sarah Ayad, Sherif Elkattawy, Chidinma Ejikeme, Abraheim Al-nasseri, Aravinda Reddy

**Affiliations:** 1 Internal Medicine, Rutgers-New Jersey Medical School / Trinitas Regional Medical Center, Elizabeth, USA; 2 Internal Medicine, New Jersey Medical School-Rutgers / Trinitas Regional Medical Center, Elizabeth, USA; 3 Internal Medicine, St. George's University School of Medicine, Elizabeth, USA; 4 Nephrology, Trinitas Regional Medical Center, Elizabeth, USA

**Keywords:** covid, aki, dialysis

## Abstract

Severe acute respiratory syndrome coronavirus 2 (SARS-CoV-2) is a highly infectious viral pathogen with high morbidity and mortality rate. The infection affects multiple organ systems leading to systemic organ failure. There is an increased incidence of acute kidney injury (AKI) in patients who become critically ill. In the critical care setting, the incidence of AKI has been variable amongst different studies. Patients with acute kidney injury who progress to renal replacement therapy are associated with worse outcomes. We describe a case of a 42-year-old male who presented with hypoxemic respiratory failure secondary to SARS-CoV-2 associated pneumonia. The patient was initially managed with the nasal cannula and then required high flow nasal cannula with worsening hypoxemic respiratory failure, requiring invasive mechanical ventilation. On top of worsening respiratory status, the patient developed new onset renal failure requiring hemodialysis.

## Introduction

Severe acute respiratory syndrome coronavirus 2 (SARS-CoV-2) has caused global economic and health implications. It has led to over 95 million infections worldwide, with over two million deaths. In the United States, it has led to 24 million infections with over 395,000 deaths. Thus far, literature has shown significant organ systems being impacted by the novel virus, with major headlines emphasizing the rapid respiratory compromise that occurs in the setting of a heightened inflammatory response, also known as the “cytokine storm.” In this report, we will shift gears and address the renal compromise in the setting of this novel virus. Multiple hypotheses have been proposed as the culprit ranging from microthrombi given hypercoagulable state in setting of heightened inflammation to direct renal tubular damage secondary to the novel virus [1-3]. This report addresses a case of a patient with no underlying medical history or chronic kidney disease who progressed to end-stage renal disease, requiring hemodialysis after coronavirus disease 2019 (COVID-19) infection.

## Case presentation

A 42-year-old male with no significant past medical history was sent from urgent care to the emergency department (ED) for evaluation of hypoxia and suspected SARS-CoV-2 infection. The patient complained of a two-day history of shortness of breath and fever. He denied any chest pain, cough, myalgia, or loss of taste or smell.

**Figure 1:**
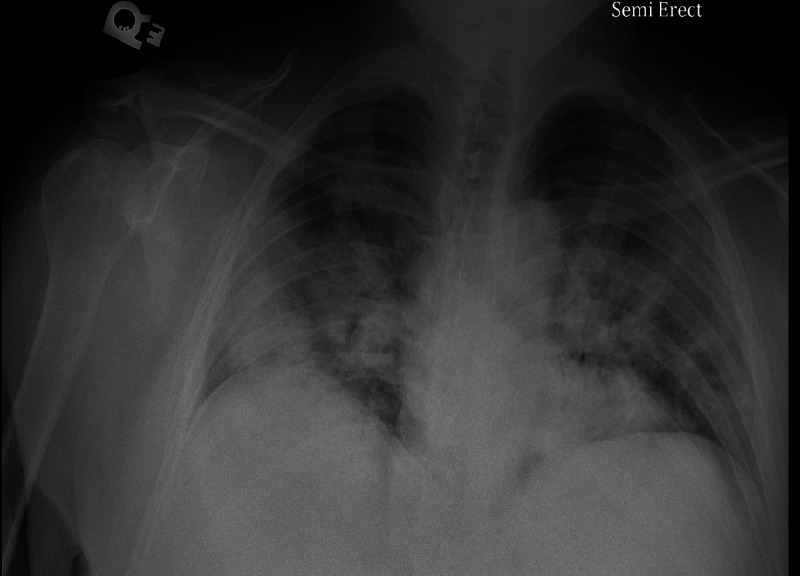
Portable chest X-ray significant for bilateral patchy airspace opacities, concerning for pneumonia

Upon arrival to the ED patient's vitals were as follows: temperature 100.5° F, pulse rate 117 beats per minute, respiratory rate 22 breaths per minute, blood pressure was 173/99 mmHg, which improved to 115/85 with no pharmacologic intervention. The patient was saturating 88% on room air at presentation and improved to 96% on 3 L of nasal cannula (NC). Chest X-ray (CXR) on presentation was significant for bilateral patchy airspace opacities concerning pneumonia, as seen in Figure 1. The patient was started on azithromycin 500 mg IV daily, ceftriaxone 1 g IV daily, dexamethasone 6 mg oral daily, enoxaparin 40 mg subcutaneous daily, and plans for remdesivir were made, and he was admitted to the general medical floor for further management.

Nasopharyngeal and oropharyngeal swabs were taken, and the patient tested positive for SARS-CoV-2 by real-time reverse-transcription polymerase chain reaction (rRT-PCR) assay. Basic metabolic panel and complete blood count laboratory values are listed in Table 1. Inflammatory markers were as follows: ferritin 2194 (23.9-336.2 ng/ml), fibrinogen 1226 (270-500 mg/dl), D-dimer 259 (0-230 ng/ml), C-reactive protein more than 20 (<10 ng/dL), lactate dehydrogenase 488 (98-192 U/L).

**Table 1:** Complete metabolic panel and complete cell count during hospitalization

	Reference range	Day 1	Day 2	Day 3	Day 4	Day 5	Day 6	Day 7	Day 8	Day 10	Day 13	Day 14	Day 15
Creatinine	0.7-1.2 mg/dl	0.81	0.63	0.63	0.65	2.25	4.76	6.85	6.39	5.53	7.32	5.82	5.81
Blood urea nitrogen (BUN)	8-20 mg/dl	11	14	19	13	23	57	90	78	73	148	121	133
Sodium	136-144 mmol/L	127	132	131	132	137	137	136	136	137	133	135	139
Potassium	3.6-5.1 mmol/L	4.2	4.4	4.1	4.2	5.4	5.1	5.2	4.7	5.5	5.8	5.6	5.6
White blood count (WBC)	4.8-10.8 k/ul	-	12.3	13.7	10.1	13.2	12.9	11.8	18.2	31.2	23.6	20.3	20.8
Hemoglobin	14-18 gm/dl	-	15.2	14.4	14.2	13.6	12.7	12.3	13.7	14.0	11.8	11.7	11.5
Platelets	130-400 K	-	253	346	371	223	221	226	269	267	205	203	213
Lymphocytes	20.5-51.1 %	-	4.3	4.8	7.6	5.6	4.0	2.9	2.4	0.8	1.7	1.1	1.7
Polys	42-75 %	-	92.7	90.2	86.5	85.8	90.3	92.2	91.5	95.9	94.6	93.4	88

On day 3, the patient was saturating 53% on ambulation, 83% on 4L NC, and 87% on a non-rebreather mask and 4L NC. On day 4, the patient continued to deteriorate and was saturating at 37% on a 100% fraction of inspired oxygen (FiO_2_) high flow oxygen nasal cannula and complained of epistaxis. The patient was intubated for type 1 respiratory failure and transferred to the intensive care unit for mechanical ventilatory support.

During the first few days of hospitalization, the patient had creatinine levels within normal limits; baseline creatinine is 0.6-.8 mg/dl. However, during his stay in the critical care unit, creatinine levels continued to rise. Table 1 shows the trend of relevant blood work. The patient’s renal insufficiency continued to worsen, requiring renal replacement therapy. The first session of hemodialysis was on day 7 of hospitalization.

**Figure 2:**
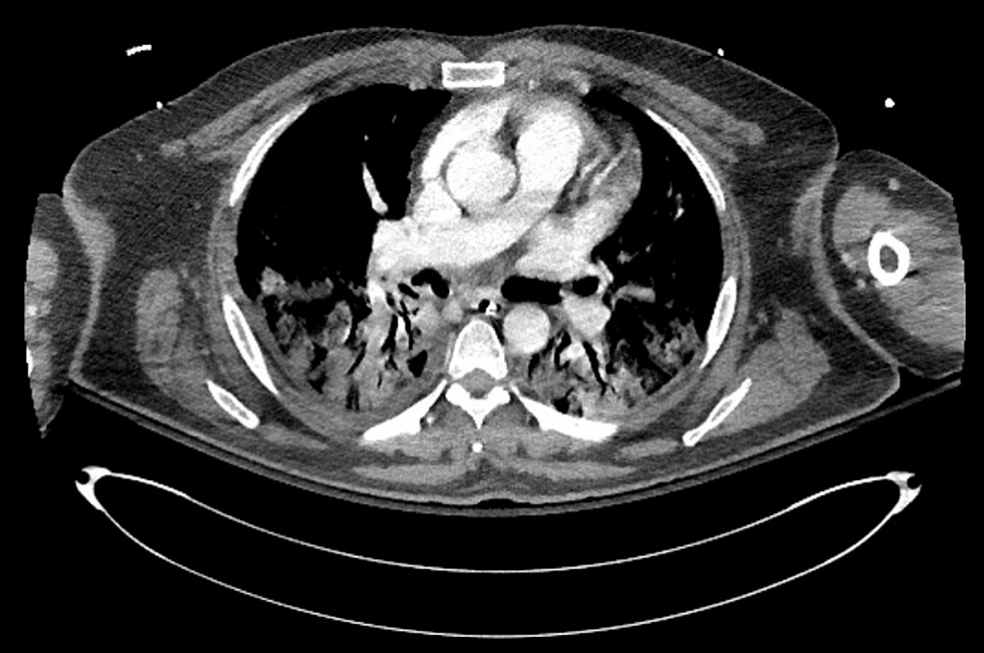
CT chest angiography significant for multiple segmental and subsegmental filling defects in the lower lobe arterial branches as well as extensive bilateral ground-glass and alveolar infiltrates

Unfortunately, the patient’s respiratory status continued to deteriorate, requiring a high FiO_2_ and positive end-expiratory pressure (PEEP). On day 8 of hospitalization, the bilateral venous duplex of the lower extremities showed evidence of acute deep vein thrombosis of the right posterior tibial, peroneal, multiple intramuscular calf veins and gastrocnemius veins and the left multiple intramuscular calf veins and gastrocnemius veins. A CAT pulmonary angiogram (CTA) showed multiple segmental and subsegmental filling defects in the lower lobe arterial branches that are highly suspicious for extensive bilateral pulmonary embolism along with extensive bilateral ground-glass and alveolar infiltrates, as seen in Figure 2. 

The patient was started on high-range heparin infusion for anticoagulation. Despite high FiO_2_, PEEP, and maximal therapy patient maintained oxygen saturation in the 70%s and 80%s. Regrettably, on day 15, the patient desaturated and eventually went into asystole. Of note, no sources of infection besides SARS-CoV-2 were detected. Full advanced cardiovascular life support protocol was performed; however, unfortunately, the return of spontaneous circulation was not achieved. 

## Discussion

Patients hospitalized for infection by SARS-CoV-2 and managed with mechanical ventilation in the intensive care unit (ICU) are at high risk for developing acute kidney injury (AKI), and consequently increased incidence of morbidity and mortality [4-8]. Complications of COVID-19-associated respiratory illnesses (i.e., acute respiratory distress syndrome [ARDS], pneumonia) necessitate positive end-expiratory pressure (PEEP) to maintain oxygen saturation levels at an appropriate level for patient survival [1]. However, PEEP can increase intrathoracic pressures leading to an increase in renal venous pressure, and consequently, reduced renal filtration [2]. Additionally, the physical dynamics of positive pressure ventilation increase sympathetic tone which activates the renin-angiotensin-aldosterone system (RAAS). Increasing levels of angiotensin-II secondary to activation of RAAS ultimately leads to decreased perfusion of the kidneys [3]. 

In addition to the complicating factors of high PEEP and mechanical ventilation, it appears that the SARS-CoV-2 itself can cause serious direct and indirect kidney injury. Current findings indicate that kidney injury is related to novel virus-mediated tubular injury as well as the heightened inflammatory response in the setting of COVID-19. The extensive inflammatory response is evidenced by the rise of inflammatory markers such as ferritin, C-reactive protein (CRP), fibrinogen, and D-dimer in patients infected with the novel virus. Furthermore, inflammation secondary to the interplay of microangiopathy and hypercoagulability can further exacerbate kidney damage [9]. 

Although many direct and/or indirect factors may contribute to AKI in patients with SARS-CoV-2, it is imperative to indicate which factors contribute the most significant effects, and thus, maximize medical management to maintain kidney function while treating for respiratory distress. Zahid et al. observed increased morbidity and mortality amongst patients with AKI and COVID-19 despite the use of renal replacement therapy (RRT). The authors noted that RRT did not enhance survival [9]. On the contrary, the use of RRT often increased the need for vasopressors requirement and other complications such as frequent clotting of dialysis catheters and line [10]. Similarly, our patient showed no improvement in renal function on hemodialysis in the setting of SARS-CoV-2 infection. Further studies are required to support these findings and elaborate on the direct and indirect causes for end-stage renal disease secondary to SARS-CoV-2 infection [10]. 

## Conclusions

The novel coronavirus has resulted in major turmoil throughout the world. The rapid clinical deterioration of patients infected with COVID-19 has caused families all over the globe grief and distress. Understanding not only the respiratory complications but also the effects on the other organ systems, in this case, the renal system will be crucial to guiding treatment. 
